# Deep learning performance on MRI prostate gland segmentation: evaluation of two commercially available algorithms compared with an expert radiologist

**DOI:** 10.1117/1.JMI.11.1.015002

**Published:** 2024-02-22

**Authors:** Erik Thimansson, Erik Baubeta, Jonatan Engman, Anders Bjartell, Sophia Zackrisson

**Affiliations:** a Lund University, Department of Translational Medicine, Diagnostic Radiology, Malmö, Sweden; b Helsingborg Hospital, Department of Radiology, Helsingborg, Sweden; c Skåne University Hospital, Department of Imaging and Functional Medicine, Malmö, Sweden; d Lund University, Department of Translational Medicine, Urology, Malmö, Sweden; e Skåne University Hospital, Department of Urology, Malmö, Sweden

**Keywords:** magnetic resonance imaging, prostate neoplasms, biopsy, radiotherapy, deep learning, prostate-specific antigen

## Abstract

**Purpose:**

Accurate whole-gland prostate segmentation is crucial for successful ultrasound-MRI fusion biopsy, focal cancer treatment, and radiation therapy techniques. Commercially available artificial intelligence (AI) models, using deep learning algorithms (DLAs) for prostate gland segmentation, are rapidly increasing in numbers. Typically, their performance in a true clinical context is scarcely examined or published. We used a heterogenous clinical MRI dataset in this study aiming to contribute to validation of AI-models.

**Approach:**

We included 123 patients in this retrospective multicenter (7 hospitals), multiscanner (8 scanners, 2 vendors, 1.5T and 3T) study comparing prostate contour assessment by 2 commercially available Food and Drug Association (FDA)-cleared and CE-marked algorithms (DLA1 and DLA2) using an expert radiologist’s manual contours as a reference standard (RSexp) in this clinical heterogeneous MRI dataset. No in-house training of the DLAs was performed before testing. Several methods for comparing segmentation overlap were used, the Dice similarity coefficient (DSC) being the most important.

**Results:**

The DSC mean and standard deviation for DLA1 versus the radiologist reference standard (RSexp) was 0.90±0.05 and for DLA2 versus RSexp it was 0.89±0.04. A paired t-test to compare the DSC for DLA1 and DLA2 showed no statistically significant difference (p=0.8).

**Conclusions:**

Two commercially available DL algorithms (FDA-cleared and CE-marked) can perform accurate whole-gland prostate segmentation on a par with expert radiologist manual planimetry on a real-world clinical dataset. Implementing AI models in the clinical routine may free up time that can be better invested in complex work tasks, adding more patient value.

## 1 Introduction

Prostate cancer is the most common male cancer. The diagnostic paradigm shift to “MRI first,”^1^^,^^2^ meaning magnetic resonance imaging (MRI) before biopsy, has increased the number of prostate MRI examinations and the number of targeted biopsies. Accurate prostate gland segmentation on MRI is crucial for ultrasound-MRI fusion biopsy planning,^3^ which is an increasingly used technique for targeted biopsies with a transrectal or transperineal approach.^4^ High-quality gland segmentation is also crucial for prostate volume calculation and prostate specific antigen (PSA) density, for focal prostate cancer therapy based on MRI findings, for brachytherapy, and for external radiation therapy planning. Several studies have evaluated deep learning (DL) algorithm performance on prostate gland segmentation and have shown good performance^5^ but, because of their single institution settings without diverse clinical MRI datasets, the results have typically not been generalizable.^6^

The number of Food and Drug Association (FDA)-cleared artificial intelligence (AI) algorithms for use in radiology is rapidly increasing and this has raised concern in the radiology community about potential risks in implementing those AI models in clinical practice outside the context in which they were developed.^7^ Several recent review articles concerning AI use in prostate MRI have noted a lack of studies validating DL algorithms’ true performance in the clinical setting^5^^,^^8^^,^^9^ and the need for larger and diverse datasets and testing of the AI models in real-life settings before routine use in clinical practice. Typically, in prior studies the authors have designed, trained, and tested a self-developed DL algorithm,^10^^,^^11^ and the majority of studies are from single-center, single-scanner settings.^6^ The MRI data require preprocessing before DL algorithm use,^12^ and most prior studies have used small test sets of under 100 patients.^6^

Even though DL prostate gland segmentation is the most commonly studied AI application, only a few commercially available DL algorithms are available (7–11; the number varies in recent review compilations).^5^^,^^13^ To our knowledge, no previous study has evaluated commercially available FDA-cleared and CE-approved DL algorithm contour quality on a diverse real-life MRI dataset.

Primary aim: to compare prostate contour assessment by two commercially available FDA-cleared and CE-marked DL algorithms to an expert radiologist’s manual contours as a reference standard.

Secondary aim: to compare prostate volume measures by two commercially available DL algorithms with expert radiologist volume measures from manual planimetry as a reference standard.

## 2 Material and Methods

### 2.1 Study Design and Population

This retrospective multicenter study was approved by the local ethics review committee at Lund University (entry no. 2014-886) and the Swedish Ethical Review Authority (entry no. 2019-03674).

**Fig. 1:**
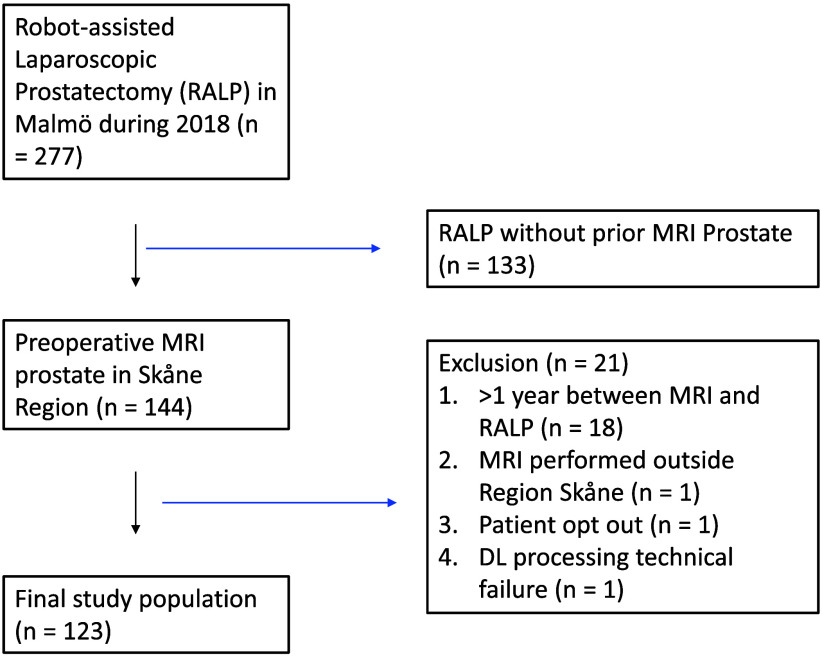
Study cohort.

Assessed for eligibility were all consecutive patients who in 2018 underwent robot-assisted radical prostatectomy at Skåne University Hospital in Malmö. Patients who had an MRI of the prostate performed less than 1 year before the date for surgery were included. Two patients were excluded due to MRI performed at a hospital outside our health care region—one patient due to patient withdrawal and one patient for DL algorithm technical reasons—resulting in the inclusion of 123 patients in the study (Fig. 1).

### 2.2 MRI Dataset and Technique

The MRI examinations were performed at seven hospitals using eight scanners, seven different scanner models from two vendors, two different field strengths (1.5 and 3T), and two T2 transaxial slice angulations. Different imaging acquisition parameters were used at different sites. All protocols included transverse, coronal, and sagittal T2-weighted turbo spin-echo images, which were used for prostate segmentation by an expert radiologist. The T2 transaxial imaging was used for DL prostate segmentation. The different scanners and T2 transaxials used are illustrated in the Supplementary Material.

### 2.3 Manual Segmentation as the Reference Standard

A consultant radiologist (JE) highly experienced in MRI/ultrasound fusion biopsy pre-processing (>800  cases) at Skåne University Hospital in Malmö Sweden, a tertiary reference center, performed prostate gland segmentation by manual planimetry, using external software MIM^®^ (version 7.1.2 MIM Software Inc, Cleveland, Ohio, United States). This workflow is used in daily clinical praxis. Time consumption was measured on a subset of the exams, which were randomly selected using a manual stopwatch. Whole-gland segmentation requires manual planimetry, a tedious and time-consuming process in which the radiologist fits a line to the prostate outer contour on all T2 transaxial slices using the coronal and sagittal slices as reference. These whole-gland segmentation contours (called Reference Standard expert, abbreviated RSexp) were used as reference standards in the study and saved as RTSS objects (a coordinate-based file format used for fusion biopsy preprocessing) and adequate for comparison with other prostate contours. JE was blinded to the DL contours.

### 2.4 Deep Learning Segmentation

**Fig. 2:**
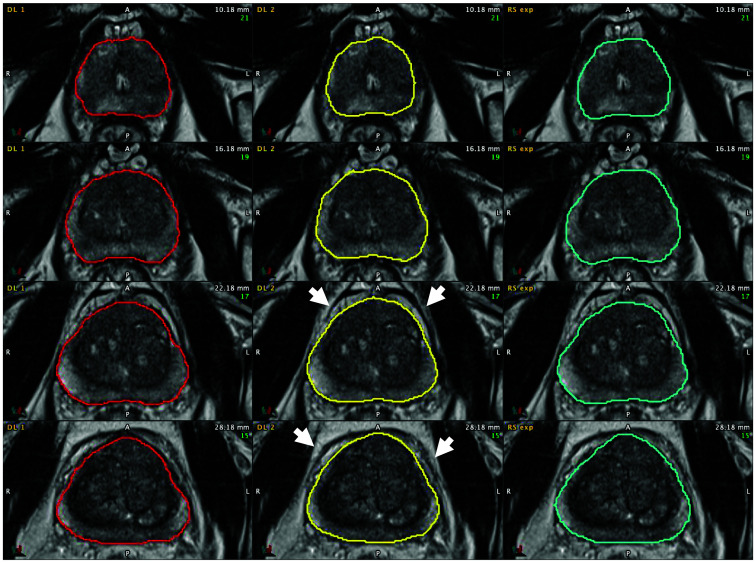
Consecutive T2 transaxials with prostate contours by planimetry. Left column: DLA1 (red contour); middle column: DLA2 (yellow contour); right column: the reference standard (turquoise contour). Both DLA1 and DLA2 succeeded in differentiating the prostate pseudocapsule from the endopelvine fascia and extraprostatic fat (white arrows in the middle column), one of the known pitfalls when performing planimetry.

Two commercially available DL algorithms were evaluated. DL algorithm 1 (called DLA1) AI-Rad Companion Prostate MR VA20A_HF02, Siemens Healthcare AG, Erlangen, Germany) is an FDA-cleared (510 [k]) and CE-marked (Class IIa–MDD) machine-learning deep-learning algorithm that uses a convolutional neural network deep image-to-image (DI2IN) network. Learning based whole gland segmentation methodology for DLA1 is described by Yang^14^ and AI model network architecture is described by Winkel.^15^ DL algorithm 2 (called DLA2) MIM contour protégé^®^ (version 7.1.7.M209-02 MIM Software Inc, Cleveland (OH), USA) is an FDA-cleared 510 [k]) and CE-marked (Class IIa – MDD) machine-learning deep-learning algorithm that uses a convolutional neural network based in the U-net architecture. The model comprises multiple layers of weights and biases that transform the input image into a segmentation mask for each structure at the final output layer. The resulting output is then post-processed to retain the single, largest connected component.^16^ For the study, the T2 transaxials were manually exported from the Picture Archive and Communications System (PACS) (Sectra IDS7, Linköping, Sweden) to DLA1 hosted on a local in-house server (A) and to DLA2 hosted on another locally hosted server (B). Neither of the algorithms was previously exposed to or trained on the images in the current study cohort. Both algorithms used non-annotated T2 transaxial images for whole-gland segmentation.^14^^–^^16^ The resulting RTSSs with DLA1 contours were exported to a server (B) where the RTSSs for DLA2 and the reference standard were localized. Prostate volumes for DLA1 and DLA2 were automatically calculated from whole-gland segmentations on server B. The above-described workflow with manual export to servers outside PACS is not used in daily practice, and for this reason its time consumption was not measured. Examples of contours from DLA1, DLA2, and RFexp are shown in Fig. 2.

### 2.5 Statistical Analysis

Two methods for comparing segmentation overlap between DL algorithms (DLAs) and reference standards were used, the DICE similarity coefficient (DSC) and the Jaccard Index. The DSC^17^ is the most commonly used measurement for prostate segmentation,^18^ and its value ranges from 0 to 1, with 0 indicating no overlap and 1 indicating perfect overlap. We also calculated the Hausdorff distance (HD), which is another widely used method^19^ for evaluating medical image segmentation. HD output is a value in mm representing how far two subsets of metric space are from each other, i.e., the greatest of all distances from a point on one contour to the closest point on the second contour. Mean and standard deviation (SD) are presented for all three methods, but since there is data skewness, we also present median and interquartile range IQR/min-max. A box plot was used to present the distribution of DSC. A paired t-test was used to compare the DSC for DLA1 and DLA2.

Bland–Altman plots were used to present method agreement when comparing prostate volume assessment by DLA1 and DLA2 with the expert radiologist as the reference standard.

Descriptive statistics were used to describe the study cohort. All statistical analyses were performed in R version 4.0.2.^20^

## 3 Results

The cohort consisted of 123 patients with a median age of 66 years (range 45 to 76 years) and median preoperative PSA of 6.90  μg/L (min 0.88; max 39).

**Fig. 3:**
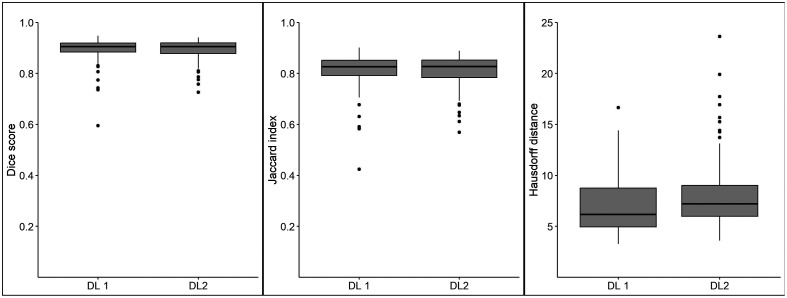
Box plot showing DICE coefficient, Jaccard Index, and HD (mm) for DLA1 and DLA2.

The DSC mean and SD for DLA1 versus the radiologist reference standard (RSexp) was 0.90±0.05 and for DLA2 versus RSexp it was 0.89±0.04. The DSC median and IQR/min-max for DLA1 was 0.91 (IQR 0.04, min-max 0.60 to 0.95) and for DL2 it was 0.91 (IQR 0.04, min-max 0.73 to 0.94). The mean Jaccard index was similar for DLA1 and DLA2 (0.81). The HD was slightly lower for DLA1 (7.1 mm) compared with DLA2 (7.9 mm). The mean, SD, IQR, and min-max for the DSC, Jaccard index, and HD are listed in Table 1 and presented as box plots in Fig. 3. A paired t-test to compare the DSCs for DLA1 and DLA2 showed no statistically significant difference (p=0.8).

**Table 1:** DSC, Jaccard index, and HD (mm) mean and SD and median and IQR/min-max for the reference standard (RSexp) and deep learning algorithms (DLA1 and DLA2).

Name	Mean	SD	Median	IQR	Min	Q1	Q3	Max
DICE RFexp vs. DLA1	0.895	0.0473	0.905	0.0355	0.595	0.884	0.920	0.948
DICE RFexp vs. DLA2	0.894	0.0386	0.905	0.0415	0.726	0.879	0.920	0.941
Jaccard RFexp vs. DLA1	0.813	0.0694	0.826	0.0595	0.424	0.792	0.851	0.901
Jaccard RFexp vs. DLA2	0.811	0.0599	0.827	0.0680	0.569	0.784	0.852	0.889
Hausdorff RFexp vs. DLA1	7.11	2.80	6.16	3.80	3.28	4.95	8.75	16.6
Hausdorff RFexp vs. DLA2	7.93	3.44	7.21	3.04	3.59	5.97	9.01	23.6

**Fig. 4:**
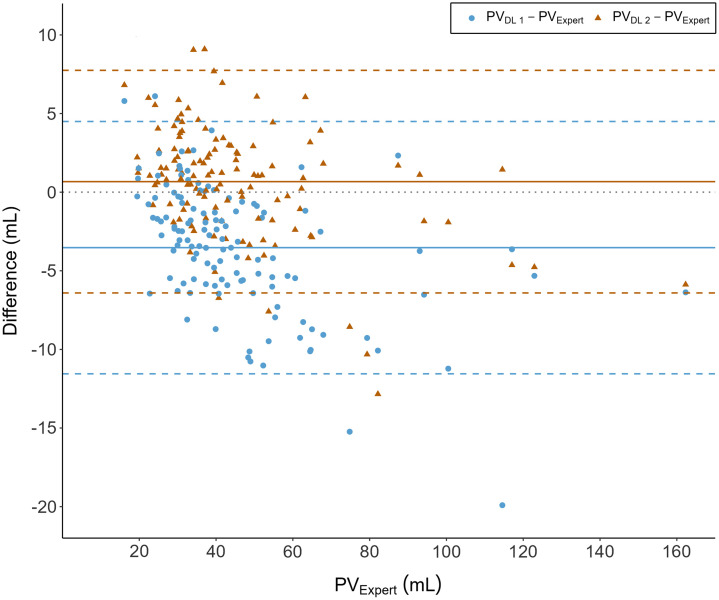
Bland–Altman plot showing method agreement for prostate volume calculation for DLA1 and DLA2 with the reference standard prostate volume calculation from manual planimetry (PVexp).

As shown in the Bland–Altman plot (Fig. 4), the observed prostate volume mean difference between DLA2 and RSexp was lower than the observed mean difference between DLA1 and RSexp (mean difference [95% limits of agreement]): DLA1, −3.53  mL (−11.55; 4.50 mL); DLA2, 0.67 mL (−6.41; 7.75 mL). DLA2 showed better precision as seen in narrower limits of agreement. DLA2 tended to overestimate the volumes of small- and medium-sized prostates, in contrast to DLA1, which tended to underestimate the volumes. Observations for planimetry by the expert radiologist were timed from start to finish in 14/123 patients, and the mean time consumption per case was 8 min and 4 s.

## 4 Discussion

In this study, we show that two commercially available FDA-cleared and CE-marked DL algorithms performed whole-gland segmentation accurately as compared with an expert radiologist’s manual planimetry as a reference standard in a diverse clinical MRI prostate dataset.

### 4.1 Whole-Gland Segmentation: Our Results in Context

In our study with 123 patients (i.e., test set n=123), the DSC mean and SD for DLA1 was 0.90±0.05, and for DLA2, it was 0.89±0.04. Our results are similar to the so-called grand challenges which serve to benchmark and validate AI models. The Promise12 challenge^18^ in 2012, the NCI-ISBI 2013^21^ challenge and the MSD^22^ challenge all showed DSC close to 0.90 (0.87–0.92). Aside from challenges, previous studies by Turkbey in 2013^23^ and Lee^11^ also demonstrated similar results (DSC 0.87–0.92). Three recent studies, however with smaller test set sizes, confirm similar agreements, Salvaggio in 2021^24^ DSC 0.90, test set n=10; Sanford in 2020^12^ DSC 0.93, test set n=29−83; and Cuocolo in 2021^25^ DSC 0.91 whole gland (Enet), test set n=105 from the ProstateX challenge. All DSCs and test set sizes are summarized in a concise table in the Supplementary Material. Thus, in our analysis of a multicenter, multiscanner clinical dataset by two commercially available DLAs, we observed the same performance that has previously been shown in segmentation challenges and recently published studies (Salvaggio single scanner, Sanford single center, and Cuocolo with 3T only).

Although both DL1 and DL2 are based on convolutional neural networks the differences in neural network architecture and training data can possibly explain at least part of those observed differences in performance between the two tested algorithms. The heterogeneity in the MRI dataset could possibly also influence the performance and explain the observed outliers in segmentation and prostate volume assessment. However, no evident motion artifacts on these specific MRI exams could be noted.

The limitations of test set size,^23^^,^^26^^,^^27^ lack of validation on external cohorts,^11^^,^^28^ and single-institution datasets^11^^,^^12^^,^^23^ were approached by Sorensen,^6^ who trained a DL model that was retrospectively tested (test set n=100 internal cases and n=56 external cases). The authors reported DSC 0.92 for ProGNet, DSC 0.85 for U-Net, and DSC 0.89 for a radiology technician, with expert segmentations used as the reference standard. In conclusion, the levels of agreement measured by DSC have remained consistently around 0.90 over the past decade. However, our study, along with Sorensen’s, demonstrates that this level of agreement holds true not only for single-center evaluations but also for multicenter real world data comparisons. In our current investigation, this level of agreement extends to two commercially available products. While our results may not be groundbreaking, they indicate that the method is robust for this relatively straightforward task and could represent a low-hanging fruit to assist and streamline the clinical workflow for radiologists.

Radiologist interreader variability was examined by Becker et al.;^29^ the author group showed an interreader DSC of 0.86 between six readers, with the highest variability found in the anatomical apex region. We have previously published interreader variability values for an experienced and an inexperienced radiologist performing manual planimetry for prostate volume calculation on an overlapping cohort. We found a small but statistically significant underestimation of prostate volumes by the inexperienced reader.^30^

In line with Bezinque et al.,^31^ who noted the lack of published performance data for DynaCad, published data on commercially available algorithms for prostate gland segmentation are still, with few exceptions, limited to conference abstracts and product white papers. The DL algorithm from the MIM contour protégé white paper^16^ reports a DSC of 0.94 from internal validation and testing. The Lucida Pi v 1.0 EuSoMII meeting presentation 2020^32^ showed a DSC of 0.92 on a test set with n=10 from PROMISE 12, and the Quantib Prostate 1.3 white paper^33^ presents internal testing of prostate volume.

### 4.2 Prostate Base and Apex: Areas with Segmentation Challenges

**Fig. 5:**
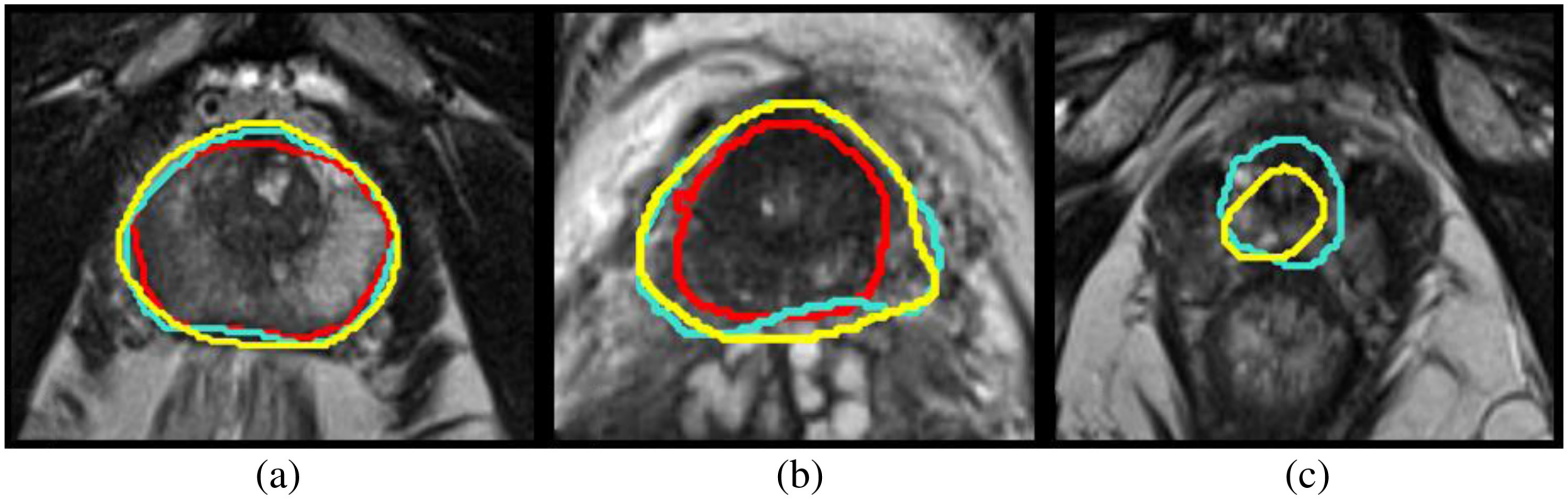
Challenges in segmentation. (a) Mid-gland, (b) base, and (c) apex. DLA1: red contour, DLA2: yellow contour, and reference standard: turquoise contour. Mid-gland, the contours are aligned; in the base and apex, larger variations in segmentation are noticeable.

To get an impression of where the algorithms face challenges, we looked into some cases with poor algorithm performance and found that the majority of differences in segmentation between algorithms and manual planimetry were found in the base and the apex of the gland. This is in line with prior studies.^24^^,^^34^ It is also our experience from clinical work with preprocessing for fusion biopsies that delineating these regions of the gland with certainty is challenging. One possible explanation for these challenges is the partial volume effect in the through-plane direction, further accentuated by relatively thick slices (3 mm) and commonly non-perpendicular slice planes with respect to the longitudinal axis of the prostate (with interindividual variation depending on the patient’s anatomical conditions). One example is shown in Fig. 5.

### 4.3 Demand for AI Model Validation and Data Overlap in Public Datasets

Several recent review articles highlight the need for adequate validation studies on AI models.^5^^,^^8^^,^^13^ The authors ask for well-curated diverse datasets,^35^ highlight the lack of large-scale multicenter prospective clinical studies,^13^ and propose that AI models be tested in real-life settings before routine use.^8^ Our current study was designed to address these concerns with a multicenter diverse dataset (including both 1.5 T and 3 T) evaluated in a real-life setting. In this context, an important concept is that training and test data must never be mixed when validating AI models.

For DL1 and probably for several other commercially available prostate segmentation algorithms, the Promise 12,^18^ ProstateX,^36^ and other public datasets have been included in the early training of the algorithm. According to the company behind DL2, no public datasets were included in its early training. Data overlap is another issue in several of the public datasets,^13^ exemplified by the inclusion of ProstateX data in the latest challenge, PICAI22.^36^ Together these emphasize the fact that validation studies ideally should be performed on clinical datasets where previous non-exposure of the algorithm can be guaranteed, as is the case in the current study.

### 4.4 Limitations

Our study has several limitations. Only two commercially available DL algorithms for whole-gland segmentation were included. According to a comprehensive comparison,^13^ 11 vendors offer segmentation, the majority bearing FDA clearance. However, it was outside the scope of the current study to include all available algorithms, since this validation on a multicenter dataset is a resource-intensive process and the dataset is limited. The size of our test set is rather small but on par with previous studies, and it was dominated by one vendor. The MRI examinations in our study are all from a radical prostatectomy cohort which does not reflect the true clinical patient mix in a radiology department. The study’s heterogeneity, with both 1.5 T and 3 T cameras, slice angulation differences, and a multicenter, multiscanner setup, improves its generalizability. Regarding the reference standard we used (manual planimetry by one expert radiologist), there is an ongoing discussion^13^ on how to obtain the best reference standard when validating AI models, since there is significant interreader variability.^27^^,^^29^ As for now, we know of no better reference standard than the one we used, and this is in line with most previous studies. We know that experts also disagree and this puts a limit to how accurately the algorithms perform, since they in principle cannot achieve better performance than the inter-expert variation. Although the performance of the two tested DL-algorithms have shown good potential to facilitate prostate volume measurements and fusion biopsy planning, the precision in segmentations do not live up to the standards required in radiation oncology where significantly lower error margins are required.

This retrospective study design should be seen as a first validation step. Future AI model validation should be designed as prospective, multivendor, multicenter trials, a planned next step for our institution in collaboration with other centers.

### 4.5 Outlook

A previous study^30^ by our group has shown that a commercially available DLA performs similarly to the radiologist-dependent standard method for prostate volume calculation. This can allow radiologist resources to be reallocated from the time-consuming and tedious task of manually measuring and calculating prostate volumes on MRI. A next step could be to use DL in the preprocessing of fusion biopsies. Although we will still have a radiologist confirming AI-processed contours (“human-in-the-loop”), this workflow clearly has the potential to save radiologists’ time. In our clinical context, we have tried this concept by comparing the time consumed in fusion biopsy pre-processing (whole-gland segmentation and lesion segmentation) by a radiologist only versus a radiologist plus AI (with whole-gland contours already in place). The trend is that substantial time saving is possible if the radiologist is presented with whole-gland contours from AI. Sorensen et al.^6^ prospectively implemented AI in the fusion biopsy process in 11 patients but did not report time measurements.

Looking forward, an AI-driven workflow including prostate gland segmentation (prostate volume, PSAD, and accurate outer contours), lesion detection, and PI-RADS characterization (including accurate lesion contouring) can be expected. However, these steps must be validated in a true clinical setting.

## 5 Conclusion

In this study, we show that two commercially available DL algorithms (FDA-cleared and CE-marked) can perform accurate whole-gland prostate segmentation on a par with expert radiologist manual planimetry on a real-world clinical dataset. Implementing AI models in the clinical routine may free up time that can be better invested by the radiologist in complex work tasks, adding more patient value.

## Supplementary Material



## Data Availability

The data utilized in this study were obtained from Skåne Region. Data are available from the authors upon request and with permission from Skåne Region.
