# Supplementary material for: Deep learning performance on MRI prostate gland segmentation: evaluation of two commercially available algorithms compared with an expert radiologist
Source: J Med Imaging (Bellingham). 2024 Feb 22;11(1):015002. doi: 10.1117/1.JMI.11.1.015002 (PMC10882278; doi:10.1117/1.JMI.11.1.015002)
Supplement: Supplementary file 1 [file JMI_011_015002_SD001.docx]

**Supplementary material**. Table illustrating the T2 transaxial characteristics.

| **Model** | **Field strength** | **angulation** | **FOV (mm)** | **aquisition matrix** | **TR/TE** | **Flip angle** | **Aquisition time (mins)** | **Slice thickness (mm)** | **Gap (mm)** | **image reconstruction (pixels)** | **Scanning resolution(mm)** |
| --- | --- | --- | --- | --- | --- | --- | --- | --- | --- | --- | --- |
| Magnetom Trio | 3T | mixed | 199*199 | 384*365 | 4190 / 104 | 132 | 04:28 | 3 | 0,6 | 0,521*0,521 | 0,518*0,545 |
| Magnetom Skyra | 3T | mixed | 200*200 | 320*310 | 4320 / 101 | 160 | 04:10 | 3 | 0,6 | 0,625*0,625 | 0,625*0,645 |
| Magnetom Prisma | 3T | mixed | 200*200 | 320*310 | 4320 / 101 | 160 | 04:10 | 3 | 0,6 | 0,625*0,625 | 0,625*0,645 |
| Magnetom Avanto Fit Kristianstad | 1,5 T | ovinklad | 180*200 | 320*275 | 7790 / 136 | 160 | 06:06 | 3 | 0 | 0,563*0,563 | 0,563*0,655 |
| Magnetom Avanto Fit Ystad | 1,5 T | ovinklad | 180*200 | 320*275 | 7500 / 102 | 160 | 04:22 | 3 | 0 | 0,563*0,563 | 0,563*0,655 |
| Signa Optima GE Hbg | 1,5 T | ovinklad | 280*280 | 352*352 | 7605 / 123 | 160 | 02:50 | 3 | 0 | 0,547*0,547 | 0795*0795 |
| Magnetom Aera Landskrona | 1,5 T | ovinklad | 200*200 | 320*275 | 7500 / 99 | 143 | 04:22 | 3 | 0 | 0,625*0,625 | 0,625*0,727 |
| Magnetom Avanto Fit Trelleborg | 1,5 T | ovinklad | 200*200 | 320*275 | 7500 / 100 | 143 | 04:22 | 3 | 0 | 0,625*0,625 | 0,625*0,727 |

**Supplementary material**. Table summarizing DICE coeefficients from previous studies.
